# Interest in genomic SNP testing for prostate cancer risk: a pilot survey

**DOI:** 10.1186/s13053-015-0032-3

**Published:** 2015-04-08

**Authors:** Michael J Hall, Karen J Ruth, David YT Chen, Laura M Gross, Veda N Giri

**Affiliations:** 1 Fox Chase Cancer Center, Philadelphia, PA USA; 2 Division of Population Science, Department of Medical Oncology, Thomas Jefferson University, Philadelphia, PA USA

**Keywords:** Genomic testing, Health literacy, Prostate cancer, Risk assessment, Single-nucleotide polymorphism

## Abstract

**Background:**

Advancements in genomic testing have led to the identification of single nucleotide polymorphisms (SNPs) associated with prostate cancer. The clinical utility of SNP tests to evaluate prostate cancer risk is unclear. Studies have not examined predictors of interest in novel genomic SNP tests for prostate cancer risk in a diverse population.

**Methods:**

Consecutive participants in the Fox Chase Prostate Cancer Risk Assessment Program (PRAP) (n = 40) and unselected men from surgical urology clinics (n = 40) completed a one-time survey. Items examined interest in genomic SNP testing for prostate cancer risk, knowledge, impact of unsolicited findings, and psychosocial factors including health literacy.

**Results:**

Knowledge of genomic SNP tests was low in both groups, but interest was higher among PRAP men (p < 0.001). The prospect of receiving unsolicited results about ancestral genomic markers increased interest in testing in both groups. Multivariable modeling identified several predictors of higher interest in a genomic SNP test including higher perceived risk (p = 0.025), indicating zero reasons for not wanting testing (vs ≥1 reason) (p = 0.013), and higher health literacy (p = 0.016).

**Conclusions:**

Knowledge of genomic SNP testing was low in this sample, but higher among high-risk men. High-risk status may increase interest in novel genomic tests, while low literacy may lessen interest.

## Background

In 2014, nearly 235,000 American men were diagnosed with prostate cancer, making it the most common noncutaneous cancer diagnosis in this group [1]. While many men will have their prostate cancer discovered at an early stage and will be curable by a combination of surgical and medical therapies, approximately 30,000 men will die from this disease [1]. African American (AA) men have the highest race-specific risk for prostate cancer, and AA race is associated with faster growth rate and more aggressive disease [2],[3]. Men with a family history of prostate cancer have a 2-to-7 fold increased risk for the disease [4],[5], with subsets at increased risk for early-onset or aggressive disease [6],[7]. Predicting risk for prostate cancer development and particularly aggressive disease would inform men to make individualized decisions for screening, prevention, and potentially disease management. Advancements in genetic and genomic testing technologies have led to the identification of several single nucleotide polymorphisms (SNPs) with association to prostate cancer [8]. These SNPs typically are common in the population and contribute modestly to increasing the risk for prostate cancer, in contrast to mutations in germ-line cancer predisposition genes such as *BRCA2* or *HOXB13* which have been shown to explain a fraction of prostate cancers related to a strong inherited predisposition [9],[10]. While the clinical utility of SNP tests to evaluate prostate cancer risk is still unclear, the presence of these markers has been shown to predict incremental increased risk for prostate cancer above family history alone [11]. Thus there is potential in the near future for growth of genomic SNP technology in clinical prostate cancer risk stratification.

In recent years, the consistent association of race to prostate cancer risk has lead researchers to investigate the relationship of known genomic markers of ancestry to prostate cancer risk. It is hypothesized that genomic markers of ancestry may add additional information in the estimation of prostate cancer risk above and beyond other genetic and/or clinical risk markers like prostate specific antigen (PSA) level [12],[13]. Indeed, many genetic association studies are designed to factor in genetic ancestry to assess the strength of association of candidate variants with prostate cancer risk [14]. Ancestral markers themselves may in fact inform risk for prostate cancer independent of other risk factors [15]. Knowledge of the association of genetic ancestry to disease risk (like cancer risk) is evolving [14],[15], and may play a meaningful role in risk assessment and risk management of diseases in the future.

Despite advancements in genomic testing, however, it is unclear how genetic testing technologies will, in the short-term, affect testing procedures like the process of informed consent, as well as patient interest and uptake of these tests. Sanger sequencing of single- or oligo-gene sites is being rapidly replaced by faster next-generation sequencing (NGS) technologies with the ability to sequence thousands of genetic loci in a single test. Clinical standards for obtaining informed consent and for disclosure of results are actively under development, but remain complicated due to the potential high volume of information produced by a single test and the uncertain clinical importance of many findings (e.g. variants of uncertain clinical significance). Studies conducted before the advent of large-scale NGS-based genomic testing have reported largely positive attitudes and high interest in genetics and genetic testing in the general public and among men at risk of prostate cancer [16]. Predictors of interest in genetic testing to stratify prostate cancer risk have included more positive attitudes toward genetic testing, better understanding and higher knowledge of genetics, stronger personal and/or family history of prostate cancer, and higher perceived risk of prostate cancer [16]-[18]. Nonetheless, few studies have not previously examined patients’ awareness of genomic SNP tests for prostate cancer risk or their interest in a genomic test if it were considered standard-of-care as part of the evaluation of prostate cancer risk. Further, other implications of genomic testing in addition to those already mentioned, including the potential for generation of ancillary or unsolicited information about genetic ancestry during testing, has also not been well studied.

In the current study, we sought to broadly explore factors associated with awareness and interest in genomic testing for prostate cancer risk in men recruited from two outpatient clinics in a tertiary cancer center setting. One group included unaffected men with racially and demographically diverse backgrounds being screened in the Prostate Cancer Risk Assessment Program, and the other included men seen in a surgical urology clinic with varied personal and family histories of prostate cancer. To better understand how psychological factors known to be important in medical decisions and specifically genetic testing decisions affect interest in genomic SNP testing within this diverse population, we also included attitudinal measures and measures of perceived cancer risk adapted from previous research, as well as measures of health literacy and numeracy in our analyses [19],[20]. Based on the previous literature, we hypothesized that perceived risk of prostate cancer, knowledge of genetics and genomic SNP tests, positive attitudes towards genomic SNP testing, and higher health literacy/numeracy skill would positively impact interest in genomic testing. Since prior literature has reported mixed results regarding interest in genetic testing and reaction to race-based medicine in African Americans [8],[21]-[26], we expected that the generation of unsolicited information about racial ancestry from a genomic test would overall negatively impact interest in testing, especially in our sample enriched in African American men.

## Materials and methods

### Recruitment

Participants were patients at Fox Chase Cancer Center and were recruited consecutively from outpatient clinics (December 2012-February 2013). Recruitment targeted two groups—1) unaffected participants undergoing prostate cancer screening in the Prostate Cancer Risk Assessment Program (PRAP) in annual follow-up, and 2) men seen in a surgical urology clinic, without exclusion for personal history or risk of prostate cancer. Men were introduced to the study by their provider, and patients were initially recruited consecutively at the end of their office visit. Recruitment of AA men was then extended in both groups to increase racial diversity until the goal of 40 subjects per group (n = 80 total) was achieved.

### Patient samples

The FCCC Prostate Cancer Risk Assessment Program (PRAP) is a screening and research program for men at high-risk for prostate cancer [27]. Eligibility criteria include men ages 35–69 with one first-degree relative with prostate cancer or two second-degree relatives with prostate cancer (same side of the family), or any AA man regardless of family history of prostate cancer, or *BRCA* mutation carrier. AA men comprise 60% of the PRAP cohort, and 60% of the entire cohort reports a family history of prostate cancer [27]. A second sample of unselected male patients was consecutively recruited from three outpatient general urology clinics (referred to as URO hereafter). These two populations were chosen to provide a broad sample of men who may have variable awareness and attitudes toward having a SNP test for hereditary prostate cancer risk, including men without prostate cancer at increased risk of prostate cancer (PRAP patients) having a yearly evaluation in a high risk clinic, and unselected men seeing their urologist for a follow-up appointment unrelated to prostate cancer risk assessment, such as evaluation for a non-oncologic issue (e.g. urinary retention) or an oncologic issue (e.g. bladder cancer).

#### Survey

The survey was administered in person by study personnel and took approximately 10 minutes to complete. Before beginning the survey questions, participants were briefly introduced to the concept of genomic SNP testing for prostate cancer risk with the following information “The following question will help us understand your awareness of single nucleotide polymorphisms (SNPs) and SNP tests. As you may already know, new blood tests that measure SNPs (pronounced SNIPS) are available over the Internet to help identify men at increased risk of prostate cancer. A SNP (single-nucleotide polymorphisms) is a marker of possible increased cancer risk present in your genes, but it does not directly cause cancer.”

##### Outcome items

Two face-valid items scored on a 5-point Likert scale were used to assess participants’ awareness of genomic SNP tests to gauge prostate cancer risk: “Before today, how aware were you of SNP tests used to help patients understand their risk of prostate cancer?” and “Were you aware that SNP tests for prostate cancer risk are available to the public over the Internet?” Two additional items assessed interest in genomic SNP testing for prostate cancer risk. The first assessed interest in a test that was considered standard-of-care:“I would be interested in having a SNP test if it was a standard part of the evaluation of risk for prostate cancer (test IS standard of care).” The second gauged interest if testing also revealed unsolicited information about ancestry “How would your interest in having a SNP test for prostate cancer risk change if the test ALSO revealed information on your ancestral or genetic origin, such as the amount or percent of your DNA that is Asian, African, or European in origin?”

##### Demographic characteristics and prostate cancer history

Age, self-identified race, marital status, and educational attainment were collected. Personal history of prostate cancer and number of first-degree relatives (FDRs) with prostate cancer was also queried.

##### Perceived risk of prostate cancer

Participants gauged their risk of developing prostate cancer relative to a man of average risk using a 5-point Likert scale (“Much lower, a little lower, same, a little higher, much higher.”

##### Attitudes towards genomic SNP testing

Reasons for wanting (n = 7 items) or not wanting (n = 7 items) a genomic SNP test for prostate cancer risk were adapted from items assessing benefits and barriers to genetic testing [28].

##### Health literacy

Three validated items [29] screened for low health literacy. Numeracy was assessed by a validated 3-item measure [30].

##### Statistical analyses

Summary statistics were examined for differences in demographic characteristics and outcomes by study group (PRAP high risk men or Urology clinic men) using chi-square tests, as we hypothesized that awareness and interest in genomic SNP testing could differ based on whether men participate in a high-risk surveillance program versus not [31],[32]. Univariate associations of demographic and psychological measures of strong interest in genomic SNP testing were tested by chi-square tests. In multivariable modeling, we developed a model including study group and psychological predictors that were associated (p < 0.10) with strong interest in SNP testing. In a second model, to reduce collinearity we omitted study group as a covariate so that we could examine other predictors which differed by study group (race, age, personal history and family history). Covariates were included in a multivariable logistic regression model, with strong interest in genomic SNP testing for prostate cancer risk (Yes/No) as the outcome. Coefficients are reported as odds ratios with 95% confidence intervals and p-values. A significance level with a two-sided α = 0.05 was used to assess the multivariable analyses.

The study was approved by the Fox Chase Cancer Center Institutional Review Board. All participants provided signed informed consent.

## Results

Eighty men, 40 from the PRAP sample (PRAP) and 40 from the unselected general urology sample (URO), completed the survey. Participant characteristics are summarized in Table 1. Participants were 40–84 years of age (mean 61.9) with PRAP group being younger than URO group (mean age 59.0 vs 64.7 years, p = 0.004). The majority of participants reported White race (66%) and were married (81%). Educational attainment was diverse--50% had a college degree but 31% had a high school diploma or less. Over half 54% (43/80) had a family history of prostate cancer, with 28% reporting one FDR and 26% at least two FDRs with prostate cancer. URO group was more than twice as likely to report a negative family history of prostate cancer. Nonetheless, a majority (62%) of the men in the URO group had a personal history of prostate cancer. PRAP recruits men at increased risk of prostate cancer—therefore, none of the 40 PRAP participants had a personal history of prostate cancer.

**Table 1:** Participant characteristics

Characteristic	All	PRAP group	Urology group	p
	N (%)	N (%)	N (%)	
**Age**				**0.014**
40-49	7 (8.8)	6 (15.0)	1 (2.5)	
50-59	20 (25.0)	14 (25.0)	6 (15.0)	
60-69	39 (48.8)	16 (40.0)	23 (57.5)	
70-84	14 (17.5)	4 (10.0)	10 (25.0)	
**Race**				**0.033**
African American	27 (33.8)	18 (45.0)	9 (22.5)	
White	53 (66.3)	22 (55.0)	31 (77.5)	
**Marital Status**				0.42
Single	9 (11.3)	4 (10.0)	5 (12.5)	
Married	65 (81.3)	31 (77.5)	34 (85.0)	
Divorced	4 (5.0)	3 (7.5)	1 (2.5)	
Widowed	2 (2.5)	2 (5.0)	0 (0.0)	
**Education**				0.69
Some HS	3 (3.8)	1 (2.5)	2 (5.0)	
High School (HS)	22 (27.5)	12 (30.0)	10 (25.0)	
Some college	15 (18.8)	9 (22.5)	6 (15.0)	
College degree	40 (50.0)	18 (45.0)	22 (55.0)	
**Internet access (yes)**	77 (96.3)	37 (92.5)	40 (100)	0.24
**Prostate cancer history**				
**Personal history**				**<0.001**
Yes	25 (31.3)	0 (0.0)	25 (62.5)	
**First-degree relatives with prostate cancer**				**0.010**
None	37 (46.3)	12 (30.0)	25 (62.5)	
1	22 (27.5)	13 (32.5)	9 (22.5)	
2+	21 (26.3)	15 (37.5)	6 (15.5)	

Bold: Results significant (p < 0.05).

The majority of men were unaware of the current availability of genomic SNP testing for prostate cancer risk (78%) [PRAP (70%) and URO (85%)(p = 0.38)] (Table 2) Surprisingly, though nearly all of the men had access to the Internet at home (96%), none of the men was aware that genomic SNP testing for prostate cancer risk was available over the Internet. However, PRAP men were more likely to agree to the statement, “I would be interested in having a SNP test if it was a standard part of the evaluation of risk for prostate cancer (test IS standard of care).” (PRAP: 75% “strongly agree”; fewer men in the URO group agreed, and 30% disagreed (30% “sort of” or “strongly” disagreed) (p < 0.001).

**Table 2:** Awareness and interest in genomic SNP testing for prostate cancer risk

Item	PRAP group	Urology group	p
	N (%)	N (%)	
**Awareness**			
**Awareness of genomic SNP testing for PC risk**			0.38
Not aware	28 (70.0)	34 (85.0)	
A little	4 (10.0)	1 (2.5)	
Somewhat	4 (10.0)	3 (7.5)	
Quite	2 (5.0)	2 (5.0)	
Very aware	2 (5.0)	0 (0.0)	
**Awareness of genomic SNP tests on the Internet**			
Not aware	40 (100.0)	38 (95.0)	0.49
Missing	0 (0.0)	2 (5.0)	
**Interest**			
**Interest in genomic SNP testing if standard-of-care**			**<0.001**
Strongly disagree	0 (0.0)	6 (15.0)	
Sort of disagree	0 (0.0)	6 (15.0)	
Neither agree nor disagree	0 (0.0)	4 (10.0)	
Sort of agree	10 (25.0)	10 (25.0)	
Strongly agree	30 (75.0)	14 (35.0)	
**Interest change if ancestry markers revealed**			0.58
No change	20 (50.0)	17 (42.5)	
Maybe more interest	12 (30.0)	17 (42.5)	
Definitely more interest	8 (20.0)	6 (15.0)	

Bold: Results significant (p < 0.05).

Refuting our hypothesis, more than half (53%) of participants expressed increased interest in genomic SNP testing when informed that the test would also reveal unsolicited information about ancestry. For PRAP men, 50% either expressed “maybe” or “definitely” more interest in genomic testing where ancestral markers of race would also be reported. Among the URO group, 58% of men expressed “maybe” or “definitely” more interest (p = 0.58). However, in the URO group, the change in interest with ancestry information was more pronounced among men with a personal history of prostate cancer versus those without (24% vs 0% expressing “definitely more interest”,p = 0.07).

### Attitudes toward SNP testing

When asked to select reasons for getting SNP testing, 64 men (80%) of the combined groups indicated “I just want to know” (Table 3). Nearly 60% of the men indicated “So I could plan for the future” and also for “To learn if my children are at risk”. Men were also asked about reasons for not getting SNP testing. Twelve men (15%) of the group indicated “I can’t do anything to prevent it”, while other reasons for not having SNP testing were indicated by only 4%-10% of the men (Table 3).

**Table 3:** Reasons for wanting and not wanting SNP testing

	n (percent)
**Reasons for wanting SNP testing**	
So I could plan for the future.	47 (58.8)
So I could make a decision about getting more health insurance.	15 (18.8)
To learn if my children are at risk.	47 (58.8)
I suspect that I am a gene carrier for cancer.	38 (47.5)
I just want to know.	64 (80.0)
To be able to take better care of myself.	40 (50.0)
To know if I need to have screening tests more often.	44 (55.0)
**Reasons for not wanting SNP testing**	
I am concerned about my emotional reaction.	3 (3.8)
I am concerned about my partner’s reaction.	4 (5.0)
I am concerned about my family’s reactions.	4 (5.0)
I just don’t want to know.	8 (10.0)
I can’t do anything to prevent it.	12 (15.0)
I would worry about how it would affect my insurance.	4 (5.0)

### Univariate analyses

URO men were less likely than PRAP men to have strong interest in SNP testing for prostate cancer risk (OR 0.18, 95% CI 0.07-0.470). Univariate associations between predictors and strong interest in genomic SNP testing are found in Table 4. The number of FDRs with prostate cancer was the only demographic or family history characteristic predictive of strong interest (p = 0.008). Interest was also associated with higher perceived risk of prostate cancer (p = 0.039) and reasons for wanting genomic SNP testing (p = 0.001), while men who indicated at least one reason for not wanting genomic SNP testing were less interested in testing (p = 0.003), as were men who had a personal history of prostate cancer (p = 0.001). Reporting needing help reading medical materials was associated with lower interest in testing (p = 0.007).

**Table 4:** Univariate associations of demographic and psychological measures with strong interest in genomic SNP testing

Predictor	Total N	Strongly agree N (%)	p
**Group**			**<0.001**
PRAP	40	30 (75.0)	
Urology,	40	147 (35.0)	
***Demographic***			
**Age**			0.10
40-64	48	30 (62.5)	
65+	32	14 (43.8)	
**Race**			0.59
African American	27	16 (59.3)	
White	53	28 (52.8)	
**Marital Status**			0.32
Married	65	34 (52.3)	
Other	15	10 (66.7)	
**Education**			0.54
High school or less	25	16 (64.0)	
Some college	15	8 (53.3)	
College or more	40	20 (50.0)	
**Internet access**			0.25
Yes	77	41 (53.2)	
No	3	3 (100.0)	
**FDRs with prostate cancer**			**0.008**
0	37	18 (48.6)	
1	22	8 (36.4)	
2+	21	18 (85.7)	
**Personal history of prostate cancer**			**0.001**
**No**	55	37 (67.3)	
**Yes**	25	7 (28.0)	
***Psychological measures***			
**Perceived risk of prostate cancer**			**0.039**
Lower	11	3 (27.3)	
Same	22	12 (54.5)	
A little higher	24	11 (45.8)	
Much higher	23	18 (78.3)	
**Reasons for wanting SNP testing**			**0.001**
0-3 reasons marked	35	12 (34.3)	
4-7 reasons marked	45	32 (71.1)	
**Reasons for not wanting SNP testing**			**0.003**
None checked	60	39 (65.0)	
At least 1 checked	20	5 (25.0)	
**Health literacy**			
*Help reading materials*			**0.007**
“None of the time”	27	9 (33.3)	
Not “none of the time”	53	35 (66.0)	
*Confidence in filling out forms*			0.78
“Extremely confident”	59	33 (55.9)	
Not “extremely confident”	21	11 (52.4)	
*Problems with understanding information*			0.34
“None of the time”	12	5 (41.7)	
Not “none of the time”	67	38 (56.7)	
**Numeracy skill**			0.22
Low numeracy (0 or 1 item correct)	16	11 (68.8)	
High numeracy (2 or 3 items correct)	64	33 (51.6)	
**Knowledge score**			
*General genetics knowledge items*			0.21
1-4 correct	10	4 (40.0)	
5-6	42	27 (64.3)	
7-8	28	13 (46.4)	
*Genomic SNP test knowledge items*			0.65
0 correct	37	19 (51.4)	
1-3	26	14 (53.8)	
4-6	17	11 (64.7)	

Bold: Results significant (p < 0.05).

### Multivariable model

Multivariable models were developed to examine the relative impact of demographic, family and personal history, and psychosocial predictors of interest in having a genomic SNP test for prostate cancer risk. The first model included study group with psychological variables that were significantly associated in the univariate analyses. In this model, study group remained a statistically significant predictor: URO men vs PRAP men, adjusted OR = 0.18 (95%CI 0.06-0.61) (Table 5). To avoid collinearity issues, we developed a second model which omitted study group and instead included age, race and personal and family history of cancer as covariates, which had been shown to differ by study group (Table 1). Results from this second model are presented in Table 6. While the small sample sizes limit interpretability of the model, the results suggest that strong family history of cancer (p = 0.026) and higher perceived risk of cancer (p = 0.036) are all positively associated with strong interest in having a genomic SNP test, while negative attitudes towards genetic testing (≥1 reason marked for not wanting genomic SNP testing)(p = 0.020), lower health literacy (needing help reading medical materials)(p = 0.017), and personal history of prostate cancer (p = 0.042) were negatively associated with interest in genomic SNP testing.

**Table 5:** Multivariable model examining clinic group and psychological predictors of strong interest in a genomic SNP test for prostate cancer risk

Predictor	OR	95% confidence interval	P
**Study group**			**0.006**
PRAP	referent		
Urology	0.18	0.06-0.61	
**Perceived risk of prostate cancer**			**0.025**
Lower	0.17	0.02-1.25	
Same	referent		
A little higher	0.25	0.06-1.15	
Much higher	2.47	0.49-12.34	
**Reasons for not wanting SNP testing**			**0.004**
None checked	referent		
At least 1 checked	0.11	0.03-0.50	
**Health Literacy**			**0.057**
*Help reading medical materials*			
“None of the time”	referent		
Not “none of the time”	0.30	0.09-1.04	

Bold: Results significant (p < 0.05).

Note: Reasons for wanting SNP testing omitted from the model due to collinearity issues.

**Table 6:** Multivariable model examining demographic, personal and family history, and psychological predictors of strong interest in a genomic SNP test for prostate cancer risk

Predictor	OR	95% confidence interval	P
**Personal history of prostate cancer**			**0.046**
No	referent		
Yes	0.16	0.03-0.97	
**Age**			0.086
40-64	referent		
65+	0.25	0.05-1.22	
**Race**			
African American	0.97	0.17-5.62	0.97
White	referent		
**FDRs with prostate cancer**			
0	referent		**0.022**
1	0.42	0.08-2.28	
2 or more	17.06	1.38-211.5	
**Perceived risk of prostate cancer**			**0.026**
Lower	0.07	0.01-0.89	
Same	referent		
A little higher	0.11	0.01-0.87	
Much higher	2.11	0.25-17.65	
**Reasons for wanting SNP testing**			0.54
0-3 reasons checked	referent		
4-7 reasons checked	1.77	0.29-10.85	
**Reasons for not wanting SNP testing**			**0.013**
None checked	referent		
At least 1 checked	0.10	0.02-0.62	
**Health Literacy**			**0.017**
*Help reading medical materials*			
“None of the time”	referent		
Not “none of the time”	0.12	0.02-0.69	

Bold: Results significant (p < 0.05).

## Discussion

Research in recent years has identified several genetic variants with strong statistical associations to prostate cancer risk [8]. The magnitude of risk associated with these SNPs is relatively modest with limited discriminative ability for cancer, leading to uncertainty regarding the clinical utility of these markers [33],[34]. However, these markers represent a fraction of the knowledge of genetic contribution to prostate cancer risk, and as more genetic determinants are uncovered there is greater likelihood of a potential clinical role in prostate cancer risk assessment. Patient interest in genomic testing will be expected to play a critical role in the near future as the clinical utility of genomic risk testing is determined. This study was performed to examine correlates of interest in genomic SNP testing in a population diverse by race, prostate cancer personal history and family history, in an effort to broadly describe factors that may be important for further investigation.

We found that there was high interest in genomic SNP testing but low awareness of the test. The strongest predictors of interest in genomic SNP testing included: being at increased risk of prostate cancer due to family history and/or AA race (PRAP), having a stronger family history of cancer, or having a higher perceived risk of cancer. Our findings complement prior studies that have reported a correlation between perceived risk, family history, and prostate cancer screening [35],[36]. However, it must be appreciated that the relationship between attitudes towards genetics, interest in testing, and actual uptake in testing is complex. Generally, the public and high-risk patients report positive attitudes towards the anticipated benefits of genetic testing and report high interest in testing, though both individual characteristics (e.g. education, worry, and perceived risk) and characteristics of the test and its results may also negatively modulate interest independent of positive attitudes [37]-[42].Whether interest in SNP testing will be associated with uptake of testing is unknown. Of important note was the low awareness of the availability of SNP testing on the Internet in this select group of patients receiving care at a tertiary care cancer center, many with a personal or familial risk of prostate cancer. This may be seen as surprising particularly given the steep rise in direct-to-consumer marketing of personal genome testing [43]. The reasons for this low awareness of Internet based genome testing in our study are unclear, but will need to be explored in follow-up studies.

Interestingly, we found that a higher knowledge score for items related to genetics and genomics did not impact men’s preferences for genomic SNP testing, either by increasing or decreasing interest. However checking even one reason for not wanting SNP testing was significantly associated with lower interest in testing. Though our sample is small, this finding suggests that interest in and uptake of a novel form of genomic testing may be reduced by specific negative views of testing. Generally positive attitudes and/or high knowledge of the role of genetics and genomics in medicine may be less influential in decisions. We also found evidence that health literacy may impact interest in testing independent of knowledge and numeracy skill. Previous research has shown health literacy to be predictive of health behaviors such as cancer screening [44] and genetic information comprehension [45], and our findings are consistent with this literature.

Our study is among the first to examine high-risk patients’ preferences toward management of unsolicited or secondary information about ancestry from genomic testing, and thus provides important preliminary insight into the potential impact commercial large-scale genomic testing could have in this population. [19] To our surprise, unlike prior research demonstrating patient displeasure with race-based personalized medicine [22],[45], our study supported a “two-for-one” concept of the positive perception of receiving additional genetic ancestry information along with genomic risk for prostate cancer. More than half of all participants expressed increased interest in genomic SNP testing if ancestry information was included and revealed, relative to interest in genomic testing in the absence of unsolicited information, while no participants reported a decline in interest (46% reported no change in interest). Research continues to explore the evolving spectrum of genetic and genomic information use and management in patients and the general public [22],[46],[47]. Studies have found the general public to be interested in gaining and/or having control over receipt of secondary information discovered during focused genetic testing, in the pharmacogenetic setting or hereditary risk setting [48]. Nonetheless, studies to better understand how cancer patients and others weigh decisions to receive this information, particularly in light of personal disease burden and other factors, remain to be performed, but early research in this area has begun to identify key attributes of unsolicited genomic information that drive patient decisions including information (i.e. disease) risk, severity, and treatability [42],[49]. Indeed some ancillary genetic information, like ancestral markers, may be more easily accepted by patients than information like increased risk of cancer because they would not be used to target treatments in a race-based approach [22],[47] and would not be associated with potentially burdensome information and the need for health behavior modifications which has been shown to be a deterrent to genetic testing [42].Equally, guidelines for best practices for consenting patients for testing that may uncover unsolicited information and for returning findings to them are still under development but hotly debated in light of the recent opinion statement published by the ACMG [50], among other reasons.

There are several limitations to note when interpreting this study. Most importantly, we assessed interest in and attitudes toward genomic SNP testing in a hypothetical format—therefore opinions may not fully reflect testing intentions and/or behaviors in a real world setting. However, men at high risk of prostate cancer or with a previous diagnosis of prostate cancer, and men who seek expert care in a high risk prostate risk clinic and/or through a tertiary care cancer center are unquestionably a relevant population who would have access to this type of testing. Secondly, the small size of our sample may have limited our ability to detect weaker associations between our outcomes and independent variables, and may have limited our ability to adequately control for the effects of the multiple independent variables included in our regression models. Future studies with larger samples of participants will be important to further investigate the independent effects of demographic and psychosocial factors on interest in and uptake of genomic testing. Finally, our finding of increased interest in genomic testing that is accompanied by unsolicited findings related to ancestral origins, while consistent with other studies, may not reflect how participants would react to unsolicited information of a different nature produced by a genomic SNP test. For example, men may express less interest in a test that revealed risk of prostate cancer in themselves but also that revealed a risk of early-onset breast cancer in their daughters, or, alternatively, a risk of early-onset dementia in family members.

In conclusion, limited health literacy and negative attitudes towards genetics may significantly dampen interest in testing. Nonetheless, patients may view unsolicited genetic information from testing in a positive light. At the present time, such insights into testing awareness of available SNP testing and interest in such testing do not automatically imply that clinicians should recommend the current SNP tests on prostate cancer risk as being clinically meaningful to our patients and to the general public. The clinical utility of SNP tests is currently unclear. Studies investigating the relationship of interest in genomic testing to action, and the psychosocial impact of unsolicited genomic information on patients in the real world setting will be valuable in the near future.

## Abbreviations

AA: African American
FCCC: Fox Chase Cancer Center
FDR: First-degree relative
NGS: Next-generation sequencing
PRAP: Prostate Risk Assessment Program
PSA: Prostate Specific Antigen
SNP: Single Nucleotide Polymorphism

## References

[1] Cancer Facts & Figures. In: Book Cancer Facts & Figures. American Cancer Society; 2013.

[2] Bigler SA, Pound CR, Zhou X (2011). A retrospective study on pathologic features and racial disparities in prostate cancer. Prostate Cancer.

[3] Powell IJ, Bock CH, Ruterbusch JJ, Sakr W (2010). Evidence supports a faster growth rate and/or earlier transformation to clinically significant prostate cancer in black than in white American men, and influences racial progression and mortality disparity. J Urol.

[4] Zeegers MP, Jellema A, Ostrer H (2003). Empiric risk of prostate carcinoma for relatives of patients with prostate carcinoma: a meta-analysis. Cancer.

[5] Kicinski M, Vangronsveld J, Nawrot TS (2011). An epidemiological reappraisal of the familial aggregation of prostate cancer: a meta-analysis. PLoS One.

[6] Hemminki K, Sundquist J, Brandt A (2011). Familial mortality and familial incidence in cancer. J Clin Oncol.

[7] Lange EM, Salinas CA, Zuhlke KA, Ray AM, Wang Y, Lu Y (2012). Early onset prostate cancer has a significant genetic component. Prostate.

[8] NIH PDQ Cancer Information Summaries. In: Book NIH PDQ Cancer Information Summaries, 2013.

[9] Liede A, Karlan BY, Narod SA (2004). Cancer risks for male carriers of germline mutations in BRCA1 or BRCA2: a review of the literature. J Clin Oncol.

[10] Ewing CM, Ray AM, Lange EM, Zuhlke KA, Robbins CM, Tembe WD (2012). Germline mutations in HOXB13 and prostate-cancer risk. N Engl J Med.

[11] Lindstrom S, Schumacher FR, Cox D, Travis RC, Albanes D, Allen NE (2012). Common genetic variants in prostate cancer risk prediction–results from the NCI Breast and Prostate Cancer Cohort Consortium (BPC3). Cancer Epidemiol Biomarkers Prev.

[12] Nassir R, Kosoy R, Tian C, White PA, Butler LM, Silva G (2009). An ancestry informative marker set for determining continental origin: validation and extension using human genome diversity panels. BMC Genet.

[13] Tian C, Hinds DA, Shigeta R, Kittles R, Ballinger DG, Seldin MF (2006). A genomewide single-nucleotide-polymorphism panel with high ancestry information for African American admixture mapping. Am J Hum Genet.

[14] Ricks-Santi LJ, Apprey V, Mason T, Wilson B, Abbas M, Hernandez W (2012). Identification of genetic risk associated with prostate cancer using ancestry informative markers. Prostate Cancer Prostatic Dis.

[15] Giri VN, Egleston B, Ruth K, Uzzo RG, Chen DY, Buyyounouski M (2009). Race, genetic West African ancestry, and prostate cancer prediction by prostate-specific antigen in prospectively screened high-risk men. Cancer Prev Res (Phila).

[16] Meisfelt S, Jones S, Cohn W, Lippert M, Haden K, Turner BL (2000). Men’s attitudes regarding testing for hereditary prostate cancer. Urology.

[17] Lanie AD, Jayaratne TE, Sheldon JP, Kardia SL, Anderson ES, Feldenbaum M (2004). Exploring the public understanding of basic genetic concepts. J Gen Counsel.

[18] Donovan KA, Tucker DC (2000). Knowledge about genetic risk for breast cancer and perceptions of genetic testing in a sociodemographically diverse sample. J Behav Med.

[19] Birmingham WC, Agarwal N, Kohlmann W, Aspinwall LG, Wang M, Bishoff J (2013). Patient and provider attitudes toward genomic testing for prostate cancer susceptibility: a mixed method study. BMC Health Serv Res.

[20] Kim SP, Knight SJ, Tomori C, Colella KM, Schoor RA, Shih L (2001). Health literacy and shared decision making for prostate cancer patients with low socioeconomic status. Cancer Invest.

[21] Forman AD, Hall MJ (2009). Influence of race/ethnicity on genetic counseling and testing for hereditary breast and ovarian cancer. Breast J.

[22] Butrick M, Roter D, Kaphingst K, Erby LH, Haywood C, Beach MC (2011). Patient reactions to personalized medicine vignettes: an experimental design. Genet Med.

[23] Rahm AK, Feigelson HS, Wagner N, Le AQ, Halterman E, Cornish N (2012). Perception of direct-to-consumer genetic testing and direct-to-consumer advertising of genetic tests among members of a large managed care organization. J Genet Couns.

[24] Kessler L, Collier A, Halbert CH (2007). Knowledge about genetics among African Americans. J Genet Couns.

[25] Suther S, Kiros GE (2009). Barriers to the use of genetic testing: a study of racial and ethnic disparities. Genet Med.

[26] Akinleye I, Roberts JS, Royal CDM, Linnenbringer E, Obisesan TO, Fasaye GA (2011). Differences between African American and white research volunteers in their attitudes, beliefs and knowledge regarding genetic testing for Alzheimer’s disease. J Genet Couns.

[27] Giri VN, Beebe-Dimmer J, Buyyounouski M, Konski A, Feigenberg SJ, Uzzo RG (2007). Prostate cancer risk assessment program: a 10-year update of cancer detection. J Urol.

[28] Lerman C, Lustbader E, Rimer B, Daly M, Miller S, Sands C (1995). Effects of individualized breast cancer risk counseling: a randomized trial. J Natl Cancer Inst.

[29] Chew LD, Griffin JM, Partin MR, Noorbaloochi S, Grill J, Snyder A (2008). Validation of screening questions for limited health literacy in a large VA outpatient population. J Gen Intern Med.

[30] Schwartz LM, Woloshin S, Black WC, Welch HG (1997). The role of numeracy in understanding the benefit of screening mammography. Ann Intern Med.

[31] Cormier L, Valeri A, Azzouzi R, Fournier G, Cussenot O, Berthon P (2002). Worry and attitude of men in at-risk families for prostate cancer about genetic susceptibility and genetic testing. Prostate.

[32] Diefenbach MA, Schnoll RA, Miller SM, Brower L (2000). Genetic testing for prostate cancer. Willingness and predictors of interest. Cancer Pract.

[33] Little J, Wilson B, Carter R, Walker K, Santaguida P, Tomiak E, et al. Multigene Panels in Prostate Cancer Risk Assessment. Evidence Report/Technology Assessment Number 209. In: Book Multigene Panels in Prostate Cancer Risk Assessment. Evidence Report/Technology Assessment Number 209. Agency for Healthcare Research and Quality (US); 2012.

[34] Park JH, Gail MH, Greene MH, Chatterjee N (2012). Potential usefulness of single nucleotide polymorphisms to identify persons at high cancer risk: an evaluation of seven common cancers. J Clin Oncol.

[35] Bloom JR, Stewart SL, Oakley-Girvans I, Banks PJ, Chang S (2006). Family history, perceived risk, and prostate cancer screening among African American men. Cancer Epidemiol Biomarkers Prev.

[36] Shavers VL, Underwood W, Moser RP (2009). Race/ethnicity, risk perception, and receipt of prostate-specific antigen testing. J Natl Med Assoc.

[37] Croyle RT, Lerman C (1993). Interest in genetic testing for colon cancer susceptibility: cognitive and emotional correlates. Prev Med.

[38] Graham ID, Logan DM, Hughes-Benzie R, Evans WK, Perras H, McAuley LM (1998). How interested is the public in genetic testing for colon cancer susceptibility? Report of a cross-sectional population survey. Cancer Prev Control.

[39] Petersen GM, Larkin E, Codori AM, Wang CY, Booker SV, Bacon J (1999). Attitudes toward colon cancer gene testing: survey of relatives of colon cancer patients. Cancer Epidemiol Biomarkers Prev.

[40] Vermeulen E, Henneman L, van El CG, Cornel MC (2014). Public attitudes towards preventive genomics and personal interest in genetic testing to prevent disease: a survey study. Eur J Public Health.

[41] Sanderson SC, Wardle J, Jarvis MJ, Humphries SE (2004). Public interest in genetic testing for susceptibility to heart disease and cancer: a population-based survey in the UK. Prev Med.

[42] Graves KD, Peshkin BN, Luta G, Tuong W, Schwartz MD (2011). Interest in genetic testing for modest changes in breast cancer risk: implications for SNP testing. Public Health Genomics.

[43] Bellcross CA, Page PZ, Meaney-Delman D (2012). Direct-to-consumer personal genome testing and cancer risk prediction. Cancer J.

[44] Peterson NB (2007). The influence of health literacy on colorectal cancer screening knowledge, beliefs and behavior. J Natl Med Assoc.

[45] Lea DH, Kaphingst KA, Bowen D, Lipkus I, Hadley DW (2010). Communicating Genetic and Genomic Information: Health Literacy and Numeracy Considerations. Public Health Genomics.

[46] Burke W, Antommaria AH, Bennett R, Botkin J, Clayton EW, Henderson GE (2013). Recommendations for returning genomic incidental findings? We need to talk!. Genet Med.

[47] Diaz VA, Mainous AG, Gavin JK, Wilson D (2014). Racial differences in attitudes toward personalized medicine. Public Health Genomics.

[48] Haga SB, O’Daniel JM, Tindall GM, Lipkus IR, Agans R (2011). Public attitudes toward ancillary information revealed by pharmacogenetic testing under limited information conditions. Genet Med.

[49] Bennette CS, Trinidad SB, Fullerton SM, Patrick D, Amendola L, Burke W (2013). Return of incidental findings in genomic medicine: measuring what patients value-development of an instrument to measure preferences for information from next-generation testing (IMPRINT)..

[50] Green RC, Berg JS, Grody WW, Kalia SS, Korf BR, Martin CL (2013). ACMG recommendations for reporting of incidental findings in clinical exome and genome sequencing. Genet Med.

